# PTEN Protein Loss in Diagnostic Prostate Biopsies Is Associated with Gleason Score Upgrading in Radical Prostatectomy

**DOI:** 10.3390/cancers18152477

**Published:** 2026-08-02

**Authors:** Nives Kolesarić, Ivan Pezelj, Igor Tomašković, Goran Štimac, Monika Ulamec, Božo Krušlin

**Affiliations:** 1 Department of Pathology and Cytology, Zabok General Hospital, 49210 Zabok, Croatia; 2 Department of Urology, Sisters of Charity Hospital, 10000 Zagreb, Croatia; pezelj.ivan@gmail.com (I.P.); igor.tomaskovic@kbcsm.hr (I.T.); goran.stimac@icloud.com (G.Š.); 3 “Ljudevit Jurak” Department of Pathology and Cytology, Sisters of Charity Hospital, 10000 Zagreb, Croatia; monika.ulamec@kbcsm.hr (M.U.); bozo.kruslin@kbcsm.hr (B.K.)

**Keywords:** prostate cancer, gleason score, upgrading, PTEN

## Abstract

**Background/Objectives:** Prostate needle biopsy often underestimates tumor aggressiveness due to limited tissue sampling, leading to Gleason score upgrading after radical prostatectomy (RP). Phosphatase and Tensin Homolog (PTEN) loss is an established tissue-based marker of adverse prostate cancer biology. This study evaluated whether reduced or absent PTEN immunoreactivity in diagnostic biopsies is associated with subsequent Gleason score and International Society of Urological Pathology (ISUP) Grade Group upgrading in RP specimens. **Methods:** This retrospective study included 85 prostate cancer patients who underwent multiparametric magnetic resonance imaging (mpMRI)-guided biopsy and subsequent RP. PTEN expression on biopsy samples was assessed via immunohistochemistry. Patients were stratified into PTEN-preserved (PTEN+, *n* = 75) and PTEN-deficient (PTEN−, *n* = 10) groups. **Results**: Upgrading occurred in 70% (7/10) of PTEN-deficient cases compared with 20% (15/75) of PTEN-preserved cases. This difference was statistically significant (two-sided Fisher’s exact *p* = 0.0024), with PTEN-deficient patients showing a 3.50-fold higher relative risk of upgrading (RR = 3.50, 95% CI: 1.91–6.43). Preoperative PSA levels (*p* = 0.91) and Prostate Imaging Reporting and Data System (PI-RADS) scores (*p* = 0.73) did not differ significantly between the groups. **Conclusions:** Reduced PTEN protein expression, as assessed by immunohistochemistry in prostate needle biopsies, was significantly associated with Gleason score/ISUP Grade Group upgrading at radical prostatectomy. PTEN immunohistochemistry warrants further evaluation as a potentially complementary tissue-based marker of biopsy undergrading. However, the observed unadjusted association does not establish PTEN immunoreactivity as an independent predictor of upgrading.

## Simple Summary

Prostate needle biopsy findings are central to treatment decisions in prostate cancer. However, biopsy samples may underestimate tumor aggressiveness because they represent only a limited portion of the tumor. This can result in Gleason score upgrading when the prostate is examined after radical prostatectomy. In this study, we evaluated whether reduced PTEN protein expression, assessed by immunohistochemistry in diagnostic needle biopsies, was associated with subsequent Gleason score upgrading in radical prostatectomy specimens. Among 85 patients, reduced or absent PTEN immunoreactivity was associated with a higher likelihood of upgrading after surgery. These findings suggest that PTEN immunohistochemistry may provide information complementary to routine histopathological assessment and may help identify patients at increased risk of biopsy undergrading.

## 1. Introduction

The clinical management of prostate cancer (PCa) is often challenging. The presence of intratumoral heterogeneity sometimes leads clinicians to stratify patients into an incorrect risk group, which can result in undertreatment or underdiagnosis [1,2,3,4,5,6]. Standard needle biopsies sample only a fraction of the prostate and the tumor itself, often leading to undergrading [7,8]. In a multifocal disease like PCa, the prostate may contain several distinct tumor nodules with different Gleason patterns [9,10,11,12,13,14,15], meaning that needle biopsies may only sample areas of the tumor with a lower Gleason pattern, failing to reflect the true tumor grade. In these cases, the biopsy suggests a low-risk tumor, but the subsequent radical prostatectomy (RP) reveals a more aggressive malignancy [7,8]. Furthermore, in some cases, the tumor may be missed altogether [16,17,18,19]. In prostate needle biopsies, the Gleason score is assigned by combining the predominant pattern with the highest-grade pattern identified, reflecting the clinical importance of even a limited high-grade component [20,21]. Additionally, the percentage of each Gleason pattern impacts the final Gleason score (GS), as the pattern present in the greater volume is considered the primary pattern, directly influencing the overall grade [20,21,22,23]. Consequently, failing to accurately determine the two most represented Gleason patterns and their ratio may lead to undergrading. For example, patients with PCa exhibiting a Gleason score of 7 (3+4) have a better prognosis than those with a score of 7 (4+3). That is one of the reasons leading to the introduction of the International Society of Urological Pathology (ISUP) Grade Group system for improved prognostic classification.

Because histological assessment of needle biopsy tissue may not fully capture the biological aggressiveness of prostate cancer, additional tissue-based biomarkers could improve preoperative risk stratification. PTEN (Phosphatase and Tensin Homolog) is a tumor suppressor gene whose loss activates the PI3K/AKT/mTOR pathway, driving cell proliferation and survival [3,24,25,26,27]; consequently, the loss of PTEN expression has been strongly associated with adverse pathological features and poorer clinical outcomes in prostate cancer. Specifically, the loss of PTEN expression suggests that the biopsy sampled the morphologically low-grade edge of a genomically aggressive tumor that likely exhibits higher-grade architecture elsewhere in the gland [4,5,25,28,29]. Although previous studies have demonstrated an association between PTEN loss and pathological upgrading, it remains insufficiently established whether this association persists in contemporary cohorts diagnosed using MRI-targeted biopsy. Therefore, the aim of this study was to evaluate whether reduced or absent PTEN immunoreactivity in prostate needle biopsies is associated with Gleason score upgrading in corresponding radical prostatectomy specimens and to assess its relationship with established clinicopathological parameters.

## 2. Materials and Methods

### 2.1. Study Design and Patient Cohort

A retrospective study was conducted using archival tissue material from the Ljudevit Jurak Clinical Department of Pathology and Cytology, Sestre Milosrdnice University Hospital Center, Zagreb, Croatia. The initial study cohort consisted of 90 randomly selected patients with prostate cancer who underwent multiparametric magnetic resonance imaging (mpMRI)-guided cognitive-targeted prostate biopsy, with additional systematic biopsy cores, between 2020 and 2024. All patients subsequently underwent radical prostatectomy (RP) at the same institution. A portion of these patients underwent mpMRI due to rising prostate-specific antigen (PSA) levels following previous negative biopsy findings.

Only cases with peripherally located tumors were included. Tumor localization was initially determined according to mpMRI findings and confirmed by histopathological examination. Patients who received neoadjuvant therapy before radical prostatectomy were excluded from the study. Additionally, five patients were excluded due to insufficient bioptic material and tissue disorganization that prevented accurate histopathological analysis, leaving a final total of 85 patients for the study.

Clinicopathological data were collected from medical records and pathology reports, including patient age, preoperative prostate-specific antigen (PSA) level, biopsy Gleason score/ISUP Grade Group, radical prostatectomy Gleason score/ISUP Grade Group, tumor localization, and the presence or absence of Gleason score upgrading. Gleason score upgrading was defined as an increase in Gleason score and/or ISUP Grade Group in the radical prostatectomy specimen compared with the corresponding diagnostic needle biopsy.

### 2.2. Histological Processing

Prostate needle biopsy samples were processed using standard histological techniques. Tissue specimens were fixed in 10% neutral buffered formalin, dehydrated through a graded series of alcohols, and embedded in paraffin blocks. Sections were cut at a thickness of 5 µm, deparaffinized in xylene, rehydrated, and stained with hematoxylin and eosin for routine histopathological evaluation.

All biopsy and radical prostatectomy specimens were reviewed by two pathologists (N.K. and B.K.) according to contemporary diagnostic criteria, and any disagreements were resolved by mutual consensus. Gleason grading was performed by identifying the predominant and highest-grade architectural patterns in biopsy specimens, while radical prostatectomy specimens were evaluated for final Gleason score and ISUP Grade Group.

### 2.3. Immunohistochemical Analysis of PTEN

PTEN expression was assessed immunohistochemically on representative tumor-containing sections from prostate needle biopsies. Biopsy material was selected by prioritizing the greatest tumor extent and the availability of sufficient residual tissue for additional sectioning. Staining was performed manually using an anti-PTEN antibody, clone Y184 (Abcam), according to the manufacturer’s instructions. PTEN positivity was assessed in the cytoplasm of tumor cells. According to the manufacturer, clone Y184 has been validated by Western blot analysis using wild-type and PTEN-knockout cell lines. Independent experimental epitope-mapping studies have localized the Y184 binding site to the C-terminal region of PTEN, approximately corresponding to amino acid residues 386–394 [30]. Non-neoplastic prostatic glands and stromal cells present on the tissue sections served as internal positive controls. No additional cell-line-based or molecular validation was performed in the present study.

### 2.4. Evaluation of PTEN Expression

Patients were stratified into two groups according to PTEN immunohistochemical expression. The PTEN-preserved group, designated as PTEN+, included tumors showing diffuse, unequivocal cytoplasmic PTEN staining in nearly all evaluated tumor cells. In practice, staining was present in 100% of tumor cells in all but one PTEN-preserved case, in which approximately 90% of tumor cells were positive.

The PTEN-deficient group, designated as PTEN−, included tumors showing either complete loss or marked heterogeneous partial loss of PTEN protein expression. In these cases, PTEN staining was present in 0% to 40% of evaluated tumor cells. No tumors showed an intermediate staining distribution between 40% and 90%. Staining intensity was not quantified as a separate continuous variable; classification was based on the presence or absence of unequivocal cytoplasmic staining in tumor cells relative to internal positive controls, consistent with the qualitative intact, heterogeneous-loss, and complete-loss framework used in previous prostate cancer studies [25,31]. Reduced or absent PTEN immunoreactivity was scored only when strong and uniform PTEN expression was present in internal positive controls, including adjacent benign prostatic glands, stromal cells, endothelial cells, and inflammatory cells. Cases without adequate internal positive-control staining were not interpreted as PTEN-deficient. Complete and heterogeneous PTEN loss were combined in the PTEN-deficient category because only 10 deficient tumors were identified, precluding a meaningful separate analysis of these patterns.

### 2.5. Statistical Analysis

Given the non-normal distribution of the data and unequal group sizes, non-parametric and exact statistical tests were applied. The Mann–Whitney U test was used to compare age, preoperative PSA levels, and Prostate Imaging Reporting and Data System (PI-RADS) scores between the PTEN-preserved and PTEN-deficient groups. Differences in biopsy ISUP Grade Group distribution according to PTEN immunohistochemical expression category were evaluated using the Fisher–Freeman–Halton exact test. The association between PTEN immunohistochemical expression category and any upgrading was assessed using the two-sided Fisher’s exact test. Upgrading was additionally categorized as no upgrading, upgrading by one ISUP Grade Group, or major upgrading, defined as an increase of two or more Grade Groups; differences in this distribution according to PTEN immunohistochemical expression category were assessed using the Fisher–Freeman–Halton exact test. A two-sided *p*-value of less than 0.05 was considered statistically significant. All statistical analyses were performed using StatSoft Statistica, version 12.

## 3. Results

Baseline clinical, pathological, and imaging characteristics according to PTEN immunohistochemical expression category are presented in Table 1. Age, preoperative PSA level, and PI-RADS score did not differ significantly between the PTEN-preserved and PTEN-deficient groups. In contrast, biopsy ISUP Grade Group distribution differed significantly according to PTEN immunohistochemical expression category (*p* = 0.005), with Grade Group 3 or 4 disease present in 80.0% of PTEN-deficient tumors compared with 25.3% of PTEN-preserved tumors.

A detailed comparison of the ISUP Grade Group distributions between the initial needle biopsy and the final radical prostatectomy specimens is presented in Table 2.

### 3.1. Immunohistochemical Assessment of PTEN Expression

**Figure 1:**
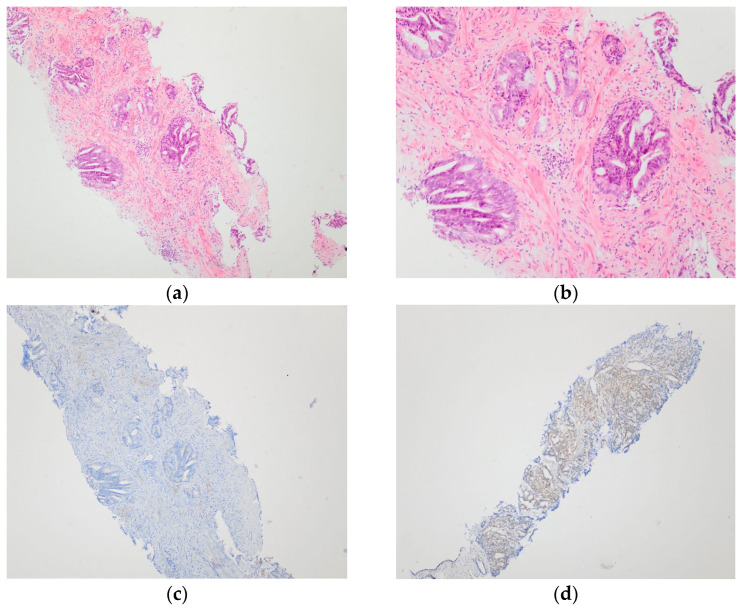
(**a**) Prostate carcinoma, needle core biopsy (hematoxylin and eosin, ×100); (**b**) prostate carcinoma, needle core biopsy (hematoxylin and eosin, ×200); (**c**) prostate carcinoma, needle core biopsy, absent PTEN immunoreactivity (×100); (**d**) prostate carcinoma, needle core biopsy, preserved PTEN immunoreactivity (×100).

Immunohistochemical analysis of the 85 diagnostic prostate needle biopsies revealed intact PTEN protein expression (PTEN-preserved group) in 75 cases (88.24%), while 10 cases (11.76%) demonstrated a heterogeneous partial or complete loss of expression (PTEN-deficient group). Representative examples of PTEN-preserved and PTEN-deficient tumors are illustrated in Figure 1.

### 3.2. Incidence of Upgrading

A significant difference in Gleason score/ISUP Grade Group upgrading was observed between the two groups (Table 3):

- PTEN-Deficient Group (*n* = 10): An upgrade occurred in 70% of cases (*n* = 7). Notably, two patients demonstrated a “major upgrade” of two or more ISUP Grade Groups (e.g., from GG 2 to GG 4).
- PTEN-Preserved Group (*n* = 75): An upgrade occurred in only 20% of cases (*n* = 15).

The incidence of upgrading differed significantly between the PTEN-deficient and PTEN-preserved groups (70% versus 20%; two-sided Fisher’s exact *p* = 0.0024). PTEN-deficient patients had a 3.50-fold higher risk of upgrading than PTEN-preserved patients (RR = 3.50, 95% CI: 1.91–6.43). When upgrading was categorized as no upgrading, upgrading by one ISUP Grade Group, or major upgrading, the distribution also differed according to PTEN immunohistochemical expression category (Fisher–Freeman–Halton exact *p* = 0.0004).

## 4. Discussion

In this retrospective cohort of 85 patients with prostate cancer diagnosed on mpMRI-guided cognitive-targeted needle biopsy and subsequently treated with radical prostatectomy, reduced or absent PTEN immunoreactivity was significantly associated with Gleason score/ISUP Grade Group upgrading. Upgrading was observed in 70% of PTEN-deficient cases compared with 20% of PTEN-preserved cases, corresponding to a 3.50-fold higher relative risk of upgrading among patients with PTEN-deficient tumors. These findings suggest that PTEN immunohistochemistry performed on diagnostic biopsy material may provide additional information beyond conventional histopathological assessment and may help identify patients at increased risk of biopsy undergrading. A distinguishing feature of the present study is the use of mpMRI-guided cognitive-targeted biopsy with additional systematic sampling. In this context, our findings extend previous observations by showing that the association between reduced or absent PTEN immunoreactivity and pathological upgrading remains detectable in a contemporary, image-guided biopsy cohort. The results should therefore be interpreted as confirmatory evidence obtained in a different diagnostic setting rather than as the first demonstration of this association.

The observed association between reduced or absent PTEN immunoreactivity and upgrading is consistent with previous evidence indicating that PTEN inactivation is linked to adverse prostate cancer biology. Lotan et al. specifically investigated PTEN protein loss in Gleason score 6 prostate needle biopsies and showed that PTEN loss was more frequent in tumors upgraded at radical prostatectomy than in non-upgraded controls [4]. In that study, PTEN protein loss was identified in 18% of upgraded cases compared with 7% of controls, and remained associated with upgrading after adjustment for age, preoperative PSA, clinical stage, and race. The reported adjusted odds ratio for upgrading was 3.04. The same study also emphasized that biopsy Gleason score may underestimate final pathology, citing an average upgrading rate of approximately 36% for apparent Gleason score 6 tumors, with reported rates ranging from 14% to 51%. In our cohort, the overall upgrading rate was 25.88%, which falls within the range reported in the literature; however, the upgrading rate in the PTEN-deficient subgroup was substantially higher, reaching 70%. Direct quantitative comparison with Lotan et al. should nevertheless be made cautiously. Their case-control study was restricted to Gleason score 6 biopsies, incorporated multivariable logistic regression, and included FISH confirmation in a subset, whereas the present cohort included several biopsy Grade Groups, used combined mpMRI-guided cognitive-targeted and systematic sampling, and assessed PTEN immunoreactivity without molecular confirmation. Our findings should therefore be regarded as supportive evidence obtained in a different diagnostic setting rather than as a direct replication of their effect estimate.

An important practical feature of PTEN immunohistochemistry is that it can be performed on routinely processed formalin-fixed, paraffin-embedded biopsy material while preserving the morphological context of the evaluated tumor focus. This allows reduced, absent, or heterogeneous staining to be assessed directly in relation to adjacent internal positive controls. In prostate cancer, PTEN immunohistochemistry is a well-established practical tissue-based assay that shows particularly high sensitivity for homozygous PTEN deletion, although concordance is lower for hemizygous deletion and a subset of IHC-loss/FISH-intact tumors remains discordant [31]. Such discordance may reflect focal genomic alterations, tumor heterogeneity, mutations, small insertions or deletions, or non-genomic mechanisms affecting PTEN expression. Sequencing studies further demonstrate that PTEN alterations may include complex structural rearrangements and point mutations, the latter potentially preserving antibody binding despite impaired protein function [32]. However, this practical applicability should not be confused with comprehensive molecular characterization. Immunohistochemistry identifies the presence of the antibody-recognized protein epitope but does not determine the underlying genetic mechanism or directly measure PTEN phosphatase activity. Accordingly, the potential role of PTEN immunohistochemistry in routine pathology would be as an accessible tissue-based adjunct rather than as a substitute for genomic testing.

The biopsy approach should be considered when interpreting the observed upgrading rate. MRI-targeted biopsy generally improves grade concordance between biopsy and radical prostatectomy, while the addition of systematic cores may further reduce undergrading by sampling tumor foci not adequately represented by targeted cores. Nevertheless, upgrading was not eliminated in the present cohort, indicating that sampling limitations and intratumoral heterogeneity may persist despite combined targeted and systematic sampling. Because the study did not include a systematic-biopsy-only comparison group, it cannot determine whether the association between PTEN immunoreactivity and upgrading differs according to biopsy technique.

Preoperative PSA levels and PI-RADS scores did not differ significantly between the PTEN-deficient and PTEN-preserved groups. However, biopsy ISUP Grade Group distribution differed significantly between the groups, with Grade Group 3 or 4 disease being more frequent among PTEN-deficient tumors. This imbalance should be considered when interpreting the association between PTEN immunoreactivity and upgrading. Because the limited number of upgrading events and PTEN-deficient cases precluded reliable multivariable adjustment, the present study cannot determine whether PTEN immunoreactivity is associated with upgrading independently of the initial biopsy Grade Group.

Our results are also in line with data from active surveillance cohorts, although direct comparison should be made cautiously because our study included patients treated with radical prostatectomy rather than men managed conservatively. In the Johns Hopkins active surveillance cohort, PTEN loss was rare among men with Gleason score 6 prostate cancer but was more frequently observed in patients who experienced early or extreme grade reclassification than in controls [33]. PTEN loss was reported in 9% of reclassified cases compared with 1.5% of controls, although this difference did not reach conventional statistical significance. These findings support the hypothesis that PTEN loss may identify a small subset of apparently low-grade tumors with higher biological potential for progression or undergrading.

Further support for the prognostic relevance of PTEN loss comes from studies evaluating adverse outcomes after radical prostatectomy. Chaux et al. showed that decreased PTEN expression was associated with increased risk of recurrence in clinically localized prostate cancer treated by radical prostatectomy [34]. In their nested case-control study, PTEN loss and markedly decreased PTEN staining were more frequent among patients who experienced recurrence, and markedly decreased PTEN expression was associated with increased recurrence risk after adjustment for established clinicopathological factors. More recently, a meta-analysis including 16 studies and 11,375 patients confirmed that PTEN loss is associated with higher ISUP Grade Group and poorer clinical outcomes, including biochemical recurrence and lethal progression. These data support the interpretation of PTEN loss as a marker of biologically more aggressive prostate cancer [3].

From a clinical perspective, PTEN testing may be particularly useful in patients whose biopsy findings suggest low-risk or favorable intermediate-risk prostate cancer. In such cases, reduced or absent PTEN immunoreactivity could alert clinicians to the possibility that the biopsy has underestimated the final tumor grade. However, PTEN immunoreactivity should not be interpreted as a standalone indication for treatment escalation. Rather, it should be integrated with established clinical, radiological, and pathological parameters, including PSA level, biopsy ISUP Grade Group, tumor extent in biopsy cores, PI-RADS score, cribriform architecture, intraductal carcinoma, and perineural invasion where available. In this way, PTEN immunohistochemistry may contribute to a more individualized assessment of patients being considered for active surveillance, focal therapy, or definitive treatment.

Nevertheless, the present study was not designed to determine whether PTEN testing changes treatment selection or improves clinical outcomes. In particular, reduced or absent PTEN immunoreactivity should not, on the basis of these findings alone, be used to exclude a patient from active surveillance or to recommend definitive treatment. Its potential value may instead lie in prompting closer consideration of all available clinical, radiological, and pathological findings when there is concern that the biopsy may underestimate tumor grade. Before PTEN immunohistochemistry can be incorporated into a formal clinical decision algorithm, prospective studies should determine whether it provides additional information beyond established predictors and whether its use meaningfully improves patient stratification.

PTEN also represents only one component of the molecular progression of prostate cancer. ERG expression was explored in the broader cohort but was not significantly associated with upgrading and was therefore not included in the focused analysis presented here. TP53 alterations, RB1 loss, Ki-67, the Decipher genomic classifier, and other molecular prognostic signatures were not assessed. Consequently, the present study cannot determine the comparative or incremental value of PTEN immunoreactivity relative to other biomarkers. Future investigations should evaluate PTEN together with broader molecular and clinicopathological profiles rather than as an isolated marker, particularly when assessing its potential contribution to preoperative risk stratification.

This study has several limitations. First, the retrospective single-institution design may limit the generalizability of the findings. Second, only 22 upgrading events were observed, and the PTEN-deficient subgroup comprised only 10 patients, which precluded a reliable multivariable analysis incorporating established clinicopathological predictors. Consequently, the present findings demonstrate an unadjusted association and should not be interpreted as evidence that PTEN immunoreactivity independently predicts upgrading. Third, PTEN expression was assessed by immunohistochemistry, which is practical and cost-effective for routine diagnostic pathology but remains a surrogate marker of underlying molecular alterations. No additional study-specific validation using PTEN-defined cell lines, Western blotting, FISH, or next-generation sequencing was performed. Although previous studies have shown good concordance between PTEN immunohistochemistry and PTEN gene deletion detected by FISH, clone Y184 recognizes an epitope within the C-terminal region of PTEN, approximately corresponding to amino acid residues 386–394 [30]. Alterations affecting this region may abolish antibody binding. Preserved immunoreactivity therefore does not exclude functionally relevant missense or catalytic mutations, post-translational alterations, or other abnormalities that retain protein expression and antibody binding. Conversely, absent staining may reflect loss or alteration of the recognized protein region and does not necessarily indicate complete genomic PTEN loss. Therefore, the present immunohistochemical findings should not be considered equivalent to a comprehensive assessment of PTEN genomic status or biological activity, and additional molecular validation would strengthen future studies. Furthermore, because PTEN immunohistochemistry was performed on selected representative biopsy material, the study could not comprehensively assess spatial heterogeneity, and focal PTEN loss in non-evaluated tumor foci may have been missed. Furthermore, the inclusion of patients with MRI-visible tumors may have introduced selection bias and may limit the generalizability of the findings. Finally, because all patients in the present cohort underwent radical prostatectomy, the results may not be directly generalizable to active surveillance populations.

The retrospective design deserves additional consideration when interpreting these findings. Although the initial cohort was randomly selected, eligibility was restricted to patients with MRI-visible peripheral tumors, adequate archival biopsy material, and subsequent radical prostatectomy at the same institution. The resulting cohort may therefore differ from the wider population undergoing prostate biopsy, including patients with MRI-invisible disease, insufficient material for ancillary testing, or conservative management. In addition, clinical variables, tissue sampling, and biomarker assessment were not collected within a prospectively standardized diagnostic protocol. The observed relationship should consequently be interpreted as an association within this selected cohort and cannot establish causality or demonstrate that PTEN testing would improve management in an unselected clinical population.

Future validation should therefore use a prospectively defined, multicentre design with standardized biopsy selection, pre-analytical processing, immunohistochemical staining, and interpretation criteria. Evaluation of multiple tumor-containing cores would allow a more systematic assessment of spatial heterogeneity, while blinded assessment by independent observers would provide formal information on reproducibility. Where sufficient tissue is available, complementary molecular methods could clarify the genomic alterations underlying preserved, reduced, or absent PTEN immunoreactivity. A larger number of upgrading events would also permit multivariable modelling incorporating established clinicopathological predictors and assessment of whether PTEN provides incremental information beyond them. Finally, prospective studies designed specifically for diagnostic evaluation would be required to estimate sensitivity, specificity, predictive values, and the clinical consequences of incorporating PTEN immunohistochemistry into treatment decisions.

Despite these limitations, our findings support the potential clinical utility of PTEN immunohistochemistry in diagnostic prostate biopsy specimens. Reduced or absent PTEN immunoreactivity was strongly associated with Gleason score/ISUP Grade Group upgrading at radical prostatectomy. Larger prospective studies are needed to validate these findings.

## 5. Conclusions

In conclusion, reduced or absent PTEN immunoreactivity in prostate needle biopsy specimens was significantly associated with Gleason score/ISUP Grade Group upgrading at radical prostatectomy. The markedly higher upgrading rate observed in PTEN-deficient tumors compared with PTEN-preserved tumors supports the potential role of PTEN immunohistochemistry as an adjunctive biomarker for identifying patients at increased risk of biopsy undergrading. Larger prospective studies are needed to validate these findings and to determine whether PTEN testing can improve preoperative risk stratification and treatment selection in prostate cancer. However, the present study does not establish that PTEN immunoreactivity predicts upgrading independently of the initial biopsy Grade Group.

## Figures and Tables

**Table 1:** Baseline clinical, pathological, and imaging characteristics according to PTEN immunohistochemical expression category.

Parameter	Total Cohort (*n* = 85)	PTEN-Preserved (*n* = 75)	PTEN-Deficient (*n* = 10)	*p*
Age, mean (range)	67.89 (55–72)	67.95 (55–72)	67.50 (59–72)	0.59
Preoperative PSA (ng/mL), Mean (range)	11.68 (4.62–21.00)	11.71 (4.62–21.00)	11.39 (7.90–16.65)	0.91
Biopsy ISUP Grade Group, n (%)				0.005
Biopsy GG1	8 (9.4%)	8 (10.7%)	0	-
Biopsy GG2	50 (58.8%)	48 (64%)	2 (20.0%)	-
Biopsy GG3	25 (29.4%)	18 (24%)	7 (70.0%)	-
Biopsy GG4	2 (2.4%)	1(1.3%)	1 (10.0%)	-
PI-RADS, mean (range)	4.13 (3–5)	4.12 (3–5)	4.20 (3–5)	0.73

Data are presented as mean (range) or *n* (%). Age, preoperative PSA level, and PI-RADS score were compared using the Mann–Whitney U test. Biopsy ISUP Grade Group distribution was compared using the Fisher–Freeman–Halton exact test.

**Table 2:** Comparison of ISUP Grade Group distributions between needle biopsy and final radical prostatectomy specimens (*n* = 85).

ISUP Grade Group	Needle Biopsy, *n* (%)	Radical Prostatectomy, *n* (%)
Grade Group 1	8 (9.41%)	0 (0.00%)
Grade Group 2	50 (58.83%)	53 (62.35%)
Grade Group 3	25 (29.41%)	26 (30.59%)
Grade Group 4	2 (2.35%)	4 (4.71%)
Grade Group 5	0 (0.00%)	2 (2.35%)

**Table 3:** Association between PTEN immunohistochemical expression category and Gleason score/ISUP Grade Group upgrading.

PTEN IHC	Upgraded, *n* (%)	Not Upgraded, *n* (%)	Total
PTEN-deficient	7 (70%)	3 (30%)	10
PTEN-preserved	15 (20%)	60 (80%)	75
Total	22 (25.88%)	63 (74.12%)	85

## Data Availability

The data presented in this study are available on request from the corresponding author. The data are not publicly available due to ethical and privacy restrictions, as they contain sensitive patient information managed within the institutional clinical database of the Sisters of Charity Hospital, Zagreb.

## Abbreviations

The following abbreviations are used in this manuscript:

AKT	Protein Kinase B
AS	Active Surveillance
FISH	Fluorescence In Situ Hybridization
GG	Grade Group
GS	Gleason Score
IHC	Immunohistochemistry
ISUP	International Society of Urological Pathology
mpMRI	Multiparametric Magnetic Resonance Imaging
mTOR	Mechanistic Target of Rapamycin
NGS	Next-Generation Sequencing
PCa	Prostate Cancer
PI3K	Phosphoinositide 3-kinase
PI-RADS	Prostate Imaging Reporting and Data System
PSA	Prostate-Specific Antigen
PTEN	Phosphatase and Tensin Homolog
RP	Radical Prostatectomy
TMA	Tissue Microarray

## References

[1] Gnanapragasam V.J., Lophatananon A., Wright K.A., Muir K.R., Gavin A., Greenberg D.C. (2016). Improving clinical risk stratification at diagnosis in primary prostate cancer: A prognostic modelling study. PLoS Med..

[2] Ruijter E.T., van de Kaa C.A., Schalken J.A., Debruyne F.M., Ruiter D.J. (1996). Histological grade heterogeneity in multifocal prostate cancer: Biological and clinical implications. J. Pathol..

[3] Kisiel F., Ferguson D., Hart C., Brown M., Oliveira P., Sachdeva A., Gardner P. (2025). Prognostic significance of PTEN loss in prostate cancer: A meta-analysis of Gleason grade and clinical outcomes. Cancers.

[4] Lotan T.L., Carvalho F.L., Peskoe S.B., Hicks J.L., Good J., Fedor H., Humphreys E., Han M., Platz E.A., Squire J.A. (2015). PTEN loss is associated with upgrading of prostate cancer from biopsy to radical prostatectomy. Mod. Pathol..

[5] Gharib A., Aziminejad A., Pourmotahari F., Kazeminejad B., Soleimani M. (2023). The rate of phosphatase and tensin (PTEN) gene expression loss in prostate cancer and its link to tumor upgrading. Urol. J..

[6] Oliveira I.S., Pontes-Junior J., Abe D.K., Crippa A., Dall’Oglio M.F., Nesrallah A.J., Leite K.R., Reis S.T., Srougi M. (2010). Undergrading and understaging in patients with clinically insignificant prostate cancer who underwent radical prostatectomy. Int. Braz. J. Urol..

[7] Lange P.H., Narayan P. (1983). Understaging and undergrading of prostate cancer: Argument for postoperative radiation as adjuvant therapy. Urology.

[8] Khoddami M., Khademi Y., Kazemi Aghdam M., Soltanghoraee H. (2016). Correlation between Gleason scores in needle biopsy and corresponding radical prostatectomy specimens: A twelve-year review. Iran. J. Pathol..

[9] Shah R.B., Bentley J., Jeffery Z., DeMarzo A.M. (2015). Heterogeneity of PTEN and ERG expression in prostate cancer on core needle biopsies: Implications for cancer risk stratification and biomarker sampling. Hum. Pathol..

[10] Haffner M.C., Zwart W., Roudier M.P., True L.D., Nelson W.G., Epstein J.I., De Marzo A.M., Nelson P.S., Yegnasubramanian S. (2021). Genomic and phenotypic heterogeneity in prostate cancer. Nat. Rev. Urol..

[11] Tolkach Y., Kristiansen G. (2018). The heterogeneity of prostate cancer: A practical approach. Pathobiology.

[12] Huang C.C., Deng F.M., Kong M.X., Ren Q., Melamed J., Zhou M. (2014). Re-evaluating the concept of “dominant/index tumor nodule” in multifocal prostate cancer. Virchows Arch..

[13] Arora R., Koch M.O., Eble J.N., Ulbright T.M., Li L., Cheng L. (2004). Heterogeneity of Gleason grade in multifocal adenocarcinoma of the prostate. Cancer.

[14] Boutros P.C., Fraser M., Harding N.J., de Borja R., Trudel D., Lalonde E., Meng A., Hennings-Yeomans P.H., McPherson A., Sabelnykova V.Y. (2015). Spatial genomic heterogeneity within localized, multifocal prostate cancer. Nat. Genet..

[15] Matsumoto K., Omura M., Takeda T., Kosaka T., Hashiguchi A., Takamatsu K., Yasumizu Y., Tanaka N., Morita S., Mizuno R. (2021). Grading of multifocal prostate cancer cases in which the largest volume and the highest grade do not coincide within one lesion. J. Urol..

[16] Serefoglu E.C., Altinova S., Ugras N.S., Akincioglu E., Asil E., Balbay M.D. (2013). How reliable is 12-core prostate biopsy procedure in the detection of prostate cancer?. Can. Urol. Assoc. J..

[17] Chelebian E., Avenel C., Järemo H., Andersson P., Wählby C., Bergh A. (2025). A clinical prostate biopsy dataset with undetected cancer. Sci. Data.

[18] Schouten M.G., van der Leest M., Pokorny M., Hoogenboom M., Barentsz J.O., Thompson L.C., Fütterer J.J. (2017). Why and where do we miss significant prostate cancer with multiparametric magnetic resonance imaging followed by magnetic resonance-guided and transrectal ultrasound-guided biopsy in biopsy-naïve men?. Eur. Urol..

[19] Eskicorapci S.Y., Baydar D.E., Akbal C., Sofikerim M., Günay M., Ekici S., Ozen H. (2004). An extended 10-core transrectal ultrasonography-guided prostate biopsy protocol improves the detection of prostate cancer. Eur. Urol..

[20] Munjal A., Leslie S.W. (2026). Gleason Score. StatPearls.

[21] Chen N., Zhou Q. (2016). The evolving Gleason grading system. Chin. J. Cancer Res..

[22] Lucia M.S., van Bokhoven A. (2012). Temporal changes in the pathologic assessment of prostate cancer. J. Natl. Cancer Inst. Monogr..

[23] Agosti V., Munari E. (2024). Histopathological evaluation and grading for prostate cancer: Current issues and crucial aspects. Asian J. Androl..

[24] Ferraldeschi R., Nava Rodrigues D., Riisnaes R., Miranda S., Figueiredo I., Rescigno P., Ravi P., Pezaro C., Omlin A., Lorente D. (2015). PTEN protein loss and clinical outcome from castration-resistant prostate cancer treated with abiraterone acetate. Eur. Urol..

[25] Jamaspishvili T., Berman D.M., Ross A.E., Scher H.I., De Marzo A.M., Squire J.A., Lotan T.L. (2018). Clinical implications of PTEN loss in prostate cancer. Nat. Rev. Urol..

[26] Ullman D., Dorn D., Rais-Bahrami S., Gordetsky J. (2018). Clinical utility and biologic implications of phosphatase and tensin homolog (PTEN) and ETS-related gene (ERG) in prostate cancer. Urology.

[27] Shorning B.Y., Dass M.S., Smalley M.J., Pearson H.B. (2020). The PI3K-AKT-mTOR pathway and prostate cancer: At the crossroads of AR, MAPK, and WNT signaling. Int. J. Mol. Sci..

[28] Wei L., Wang J., Lampert E., Schlanger S., DePriest A.D., Hu Q., Gomez E.C., Murakami M., Glenn S.T., Conroy J. (2017). Intratumoral and intertumoral genomic heterogeneity of multifocal localized prostate cancer impacts molecular classifications and genomic prognosticators. Eur. Urol..

[29] Yoshimoto M., Ding K., Sweet J.M., Ludkovski O., Trottier G., Song K.S., Joshua A.M., Fleshner N.E., Squire J.A., Evans A.J. (2013). PTEN losses exhibit heterogeneity in multifocal prostatic adenocarcinoma and are associated with higher Gleason grade. Mod. Pathol..

[30] Mingo J., Luna S., Gaafar A., Nunes-Xavier C.E., Torices L., Mosteiro L., Ruiz R., Guerra I., Llarena R., Angulo J.C. (2019). Precise definition of PTEN C-terminal epitopes and its implications in clinical oncology. npj Precis. Oncol..

[31] Lotan T.L., Wei W., Ludkovski O., Morais C.L., Guedes L.B., Jamaspishvili T., Lopez K., Hawley S.T., Feng Z., Fazli L. (2016). Analytic validation of a clinical-grade PTEN immunohistochemistry assay in prostate cancer by comparison with PTEN FISH. Mod. Pathol..

[32] Murphy S.J., Karnes R.J., Kosari F., Castellar B.E.R., Kipp B.R., Johnson S.H., Terra S., Harris F.R., Halling G.C., Klein J.L. (2016). Integrated analysis of the genomic instability of PTEN in clinically insignificant and significant prostate cancer. Mod. Pathol..

[33] Tosoian J.J., Guedes L.B., Morais C.L., Mamawala M., Ross A.E., De Marzo A.M., Trock B.J., Han M., Carter H.B., Lotan T.L. (2019). PTEN status assessment in the Johns Hopkins active surveillance cohort. Prostate Cancer Prostatic Dis..

[34] Chaux A., Peskoe S.B., Gonzalez-Roibon N., Schultz L., Albadine R., Hicks J., De Marzo A.M., Platz E.A., Netto G.J. (2012). Loss of PTEN expression is associated with increased risk of recurrence after prostatectomy for clinically localized prostate cancer. Mod. Pathol..

